# Administration route-dependent vaccine efficiency of murine dendritic cells pulsed with antigens

**DOI:** 10.1054/bjoc.2001.1801

**Published:** 2001-06

**Authors:** N Okada, M Tsujino, Y Hagiwara, A Tada, Y Tamura, K Mori, T Saito, S Nakagawa, T Mayumi, T Fujita, A Yamamoto

**Affiliations:** Department of Biopharmaceutics, Kyoto Pharmaceutical University, Misasagi, Yamashina-ku, Kyoto 607-8414, Japan; Department of Biopharmaceutics, Graduate School of Pharmaceutical Sciences, Osaka University, 1-6 Yamadaoka, Suita, Osaka 565-0871, Japan

**Keywords:** dendritic cell, administration route, vaccine efficiency, cytotoxic T lymphocyte, migration

## Abstract

Dendritic cells (DCs) loaded with tumour antigens have been successfully used to induce protective tumour immunity in murine models and human trials. However, it is still unclear which DC administration route elicits a superior therapeutic effect. Herein, we investigated the vaccine efficiency of DC2.4 cells, a murine dendritic cell line, pulsed with ovalbumin (OVA) in the murine E.G7-OVA tumour model after immunization via various routes. After a single vaccination using 1 × 10^6^OVA-pulsed DC2.4 cells, tumour was completely rejected in the intradermally (i.d.; three of four mice), subcutaneously (s.c.; three of four mice), and intraperitoneally (i.p.; one of four mice) immunized groups. Double vaccinations enhanced the anti-tumour effect in all groups except the intravenous (i.v.) group, which failed to achieve complete rejection. The anti-tumour efficacy of each immunization route was correlated with the OVA-specific cytotoxic T lymphocyte (CTL) activity evaluated on day 7 post-vaccination. Furthermore, the accumulation of DC2.4 cells in the regional lymph nodes was detected only in the i.d.-and s.c.-injected groups. These results demonstrate that the administration route of antigen-loaded DCs affects the migration of DCs to lymphoid tissues and the magnitude of antigen-specific CTL response. Furthermore, the immunization route affects vaccine efficiency. © 2001 Cancer Research Campaign http://www.bjcancer.com


British Journal of Cancer (2001) 84(11), 1564–1570
© 2001 Cancer Research Campaign
doi: 10.1054/ bjoc.2001.1801, available online at http://www.idealibrary.com on IDEAL®

http://www.bjcancer.com

### Administration route-dependent vaccine efficiency of murine dendritic cells pulsed with antigens

**N Okada[1], M Tsujino[1], Y Hagiwara[1], A Tada[1], Y Tamura[1], K Mori[1], T Saito[1], S Nakagawa[2], T Mayumi[2], T Fujita[1] and A Yamamoto[1]**

[1]Department of Biopharmaceutics, Kyoto Pharmaceutical University, Misasagi, Yamashina-ku, Kyoto 607-8414, Japan; [2]Department of Biopharmaceutics, Graduate School of Pharmaceutical Sciences, Osaka University, 1-6 Yamadaoka, Suita, Osaka 565-0871, Japan

**Summary** Dendritic cells (DCs) loaded with tumour antigens have been successfully used to induce protective tumour immunity in murine models and human trials. However, it is still unclear which DC administration route elicits a superior therapeutic effect. Herein, we investigated the vaccine efficiency of DC2.4 cells, a murine dendritic cell line, pulsed with ovalbumin (OVA) in the murine E.G7-OVA tumour model after immunization via various routes. After a single vaccination using $1 \times 10^6$ OVA-pulsed DC2.4 cells, tumour was completely rejected in the intradermally (i.d.; three of four mice), subcutaneously (s.c.; three of four mice), and intraperitoneally (i.p.; one of four mice) immunized groups. Double vaccinations enhanced the anti-tumour effect in all groups except the intravenous (i.v.) group, which failed to achieve complete rejection. The anti-tumour efficacy of each immunization route was correlated with the OVA-specific cytotoxic T lymphocyte (CTL) activity evaluated on day 7 post-vaccination. Furthermore, the accumulation of DC2.4 cells in the regional lymph nodes was detected only in the i.d.-and s.c.-injected groups. These results demonstrate that the administration route of antigen-loaded DCs affects the migration of DCs to lymphoid tissues and the magnitude of antigen-specific CTL response. Furthermore, the immunization route affects vaccine efficiency. © 2001 Cancer Research Campagin http://www.bjcancer.com

**Keywords**: dendritic cell; administration route; vaccine efficiency; cytotoxic T lymphocyte; migration

$CD8^+$ cytotoxic T lymphocytes (CTLs) kill neoplastic cells through the recognition of short peptide epitopes (8–12 amino acids long) presented by major histocompatibility complex (MHC) class I molecules that are expressed on the target cell surface (Kronenberg et al, 1986; Henkart, 1994). Although the recognition of peptide-MHC class I complexes is sufficient to trigger tumour cell lysis, priming of CTL responses requires the presentation of the relevant antigen by professional antigen-presenting cells (APCs) capable of providing co-stimulation. Vaccines and immunotherapies that preferentially prime this component of the immune response are critical for establishing effective host tumour immunity.

Dendritic cells (DCs) are professional APCs playing a key function in the immune system as initiators of T-cell responses against microbial pathogens and tumours (Steinman, 1991; Banchereau and Steinman, 1998). Immature DCs capture and process antigens in peripheral tissues and begin to mature. The mature DCs migrate to lymphoid organs, where they stimulate naïve T cells through the signals of both MHC molecules presenting antigen-peptides and costimulatory molecules (Austyn et al, 1988; Rock, 1996). Therefore, immunization using DCs loaded with tumour-associated antigens (TAAs) is potentially a powerful method of inducing anti-tumour immunity. In fact, a number of studies have shown that immunization of mice by DCs pulsed with TAAs can prime a tumour-specific CTL response and engender protective tumour immunity (Mayordomo et al, 1995; Celluzzi et al, 1996; Nair et al, 1997). These results provide a promising experimental basis for developing clinical trials of the anti-tumour therapy using DCs (Hsu et al, 1996; Nestle et al, 1998; Lodge et al, 2000). However, further investigation of DC-based immunotherapy is required to: (1) establish efficient methods for collection and expansion of large numbers of DC precursors (Romani et al, 1994; Bernhard et al, 1995); (2) improve methods for induction and purification of effective DCs (Fearnley et al, 1997; Brossart et al, 1998; Lutz et al, 1999; Muller et al, 2000); (3) identify and select antigens (e.g. peptides, proteins, DNA, mRNA, or tumour lysate) that can elicit specific and effective immune responses (Ashley et al, 1997; Nestle et al, 1998; Philip et al, 2000); (4) develop methods for efficient introduction of antigens into DCs (Nair et al, 1992; Arthur et al, 1997; Gong et al, 1997; Tillman et al, 1999); (5) identify an optimized DC-immunization protocol (Eggert et al, 1999; Fallarino et al, 1999; Lappin et al, 1999); and (6) confirm the safety of a DC-vaccination (Roskrow et al, 1999; Ludewig et al, 2000).

In the present research, we examined the usefulness of Lipofectin™, a commercially available cationic liposome for introducing plasmid DNA, for the introduction of exogenous antigens into DCs by evaluating antigen presentation on MHC class I molecules. In order to identify the DC-immunization protocol that exhibits the most superior anti-tumour effect, we investigated the vaccine efficiency of DC2.4 cells, a murine dendritic cell line, pulsed with ovalbumin (OVA) after i.v., i.p., i.d., or s.c. immunization in the murine E.G7-OVA tumour model. In addition, we investigated the effect of OVA-pulsed DC2.4 cells-immunization route on the induction of OVA-specific CTL response and on the migration of DC2.4 cells to regional lymph nodes.

*Received 30 October 2000*
*Revised 5 March 2001*
*Accepted 6 March 2001*

*Correspondence to: N Okada*

### MATERIALS AND METHOODS

### Cells and animals

DC2.4 cells (H-$2^b$) and RF33.70 cells (T-T hybridoma against $OVA^+$ H-$2K^b$) were generously provided by Dr K L Rock (Department of Pathology, University of Massachusetts Medical School, MA, USA). DC2.4 cells, a DC line established from bone marrow cells of C57BL/6 mice by infection with a retrovirus encoding *v-myc* and *v-raf* (Shen et al, 1997), were grown in complete RPMI 1640 medium (10% fetal bovine serum, 2 mM L-glutamine, 100 µg/ml streptomycin, and 100 U/ml penicillin) supplemented with non-essential amino acid (100 µM) and 2-mercaptoethanol (2-ME: 50 µM). RF33.70 cells (Rock et al, 1990; Shen et al, 1997) were maintained in complete RPMI 1640 medium supplemented with 50 µM 2-ME. CTLL-2 cells, which proliferate specifically in response to interleukin-2 (IL-2) (Harding, 1994), were maintained in complete RPMI 1640 medium supplemented with 50 µM 2-ME and 10 U/ml murine recombinant IL-2 (Pepro Tech EC LTD., London, England). E.G7-OVA tumour cells (H-$2^b$; OVA-transfectant of EL4 murine thymoma cells) were maintained in RPMI 1640 medium supplemented with 10% fetal bovine serum, 50 µM 2-ME and 400 µg/ml G418. EL4 cells and YAC-1 cells, which are highly sensitive to natural killer cells (Cikes et al, 1973), were maintained in complete RPMI 1640 medium supplemented with 50 µM 2-ME.

Female C57BL/6 mice, aged 7–8 weeks, were purchased from SLC Inc. (Hamamatsu, Japan) and held under specified pathogen-free conditions. All of the experimental procedures carried out were in accordance with the Kyoto Pharmaceutical University guidelines on animal experiments and the United Kingdom Co-ordinating Committee on Cancer Research (UKCCCR) guidelines for the welfare of animals in experimental neoplasia.

### Antigen presentation assay

DC2.4 cells were seeded in a 96-well flat-bottom culture plate at a density of $1 \times 10^4$ cells/well and cultured overnight. Lipofectin™ (Life Technologies, Tokyo, Japan), composed of N-[1-(2,3-dioleyloxy)propyl]-N, N, N-trimethylammonium chloride (DOTMA) and dioleoyl phosphatidylethanol amine (DOPE), was mixed at a final concentration of 10 µg/ml with various concentrations of OVA (Sigma Chemical Co., St Louis, MO, USA) in Opti-MEM (Life Technologies), and incubated for 20 min at room temperature. Each well was washed twice with Opti-MEM, and then the cells were incubated with various concentrations of soluble OVA or OVA-Lipofectin complex in 100 µl of Opti-MEM. After 5-h incubation at 37˚C, each well was washed twice and incubated in the absence or presence of mitomycin C (MMC: 50 µg/ml in Opti-MEM) at 37˚C for 30 min. After washing with Opti-MEM, OVA-pulsed DC2.4 cells were co-cultured with specific T-T hybridoma against $OVA^+$ H-$2K^b$, RF33.70 cells (Rock et al, 1990; Shen et al, 1997). The response of stimulated RF33.70 cells ($1 \times 10^5$ cells/well) was assessed by determining IL-2 released into an aliquot of complete RPMI 1640 medium (200 µl) at 37˚C. Briefly, the culture medium was collected after 20-h co-culturing of OVA-pulsed DC2.4 cells and RF33.70 cells. Serially diluted culture medium was added to the CTLL-2 cells seeded on 96-well flat-bottom culture plates at a density of $5 \times 10^3$ cells/well. Two days later, the number of viable CTLL-2 cells was evaluated by MTT assay (Mosmann, 1983). The concentration of IL-2 released from RF33.70 cells was calculated from a standard curve obtained from the MTT activity of CTLL-2 cells cultured in the presence of various concentrations of murine recombinant IL-2.

### Loading OVA into DC2.4 cells using lipofectin

Lipofectin was mixed at a final concentration of 10 µg/ml with OVA at a final concentration of 1 mg/ml in Opti-MEM, and incubated for 20 min at room temperature. The OVA-Lipofectin mixture was added to a DC2.4 cell pellet ($1 \times 10^6$ cells/ml) that was previously washed twice in Opti-MEM, and the final solution incubated at 37˚C in a water bath with occasional agitation for 5 h. After washing with phosphate-buffered saline (PBS), the cells were treated with MMC (50 µg/ml in Opti-MEM) at 37˚C for 30 min in order to inhibit proliferation. OVA-pulsed DC2.4 cells were then washed twice and resuspended with PBS prior to immunization.

### Tumour rejection assay

C57BL/6 mice were immunized once or twice over a 1-week interval with OVA-pulsed DC2.4 cells. Cells ($1 \times 10^6$ cells/100 µl/mouse) were injected into the lateral tail vein (i.v.), peritoneum (i.p.), or right flank (i.d. or s.c.). One week after the final vaccination, $1 \times 10^6$ E.G7-OVA tumour cells were inoculated into the left flank. Tumour size was assessed three times a week using microcalipers and was expressed as tumour volume, calculated by the following formula (Janik et al, 1975): tumour volume ($mm^3$) = (major axis) × (minor axis)$^2$ × 0.5236. Mice with tumour sizes > 2.5 cm or tumour volume > 2500 $mm^3$ were euthanized. On day 60 after tumour challenge, all survivors were euthanized.

### Europium-release assay for detecting OVA-specific CTLs

C57BL/6 mice were immunized once with OVA-pulsed or non-pulsed DC2.4 cells ($1 \times 10^6$ cells). At one week after immunization, spleens were excised and single cells suspensions were prepared. The splenocytes were re-stimulated in vitro with E.G7-OVA cells pre-treated with MMC (50 µg/ml in Opti-MEM) at 37˚C for 30 min, at an effector:stimulator ratio of 10:1 in complete RPMI 1640 medium. Five days after re-stimulation, europium (Eu)-labeling of target cells (E.G7-OVA cells, EL4 cells, and YAC-1 cells) and Eu-release assay were performed according to the methods of Volgmann et al (1989). In brief, target cells (5–10 × $10^6$) were labeled with Eu chelate (Eu-diethylenetriamine pentaacetate) for 20 min at 4˚C. Eu-labeled targets ($1 \times 10^4$ cells) were incubated with serially diluted effector cells at various effector:target (E:T) ratios in 100 µl of complete RPMI 1640 medium on 96-well U-bottomed culture plates. The plates were centrifuged at 500 × g for 3 min and incubated at 37˚C for 4 h. The addition of 150 µl of an enhancement solution (Amersham Pharmacia Biotech, Tokyo, Japan) containing β-naphthoyltrifluoroacetone to 50 µl of culture supernatant aliquots leads to the formation of a highly fluorescent Eu-chelate that can be measured with a time-resolved fluorometer (Ex: 340 nm, Em: 612 nm; SPECTARAFLUOR Plus, TECAN, Tokyo, Japan). Specific cytotoxic activity was determined using the following formula: specific lysis (%) = [(experimental Eu-release – spontaneous Eu-release) / (maximum Eu-release – spontaneous Eu-release)] × 100. Spontaneous Eu-release of the target cells was < 10% of maximum Eu-release by detergent in all assays. SEM was less than 5% of triplicate cultures.

© 2001 Cancer Research Campaign

### Estimation of DC2.4 cells migration to the lymphoid tissues by polymerase chain reaction (PCR)

C57BL/6 mice were injected with $1 \times 10^7$ OVA-pulsed DC2.4 cells via various routes including i.v. (lateral tail vein), i.p., i.d. (right flank), and s.c. (right flank). At 48 h after injection, mice were sacrificed and thymus, spleen, Peyer's patches and lymph nodes (cervical, brachial, axillary, mesenterial, renal, lumbal, pygal, inguinal, and ischial) were excised and genomic DNA was isolated from these tissues. Because *v-myc* is inserted in genomic DNA of DC2.4 cells (Shen et al, 1997), we attempted to estimate the migration of DC2.4 cells to the lymphoid tissues by amplification of the *v-myc* gene using PCR. The primers for *v-myc* amplification were designed based on GeneBank data for *v-myc* derived from avian myelocytomatosis virus (accession No. V01173) and had the following sequence: forward, 5′-AACGAGTCTGAATCCAGC AC-3′; reverse, 5′-TGCAAGACAGAGAGGTTAGC-3′. Genomic DNA isolated from DC2.4 cells was used as a positive control. The amplification conditions were as follows: after denaturation at 94˚C for 2 min, denaturation at 94˚C for 45 s, annealing at 52˚C for 45 s, and extension at 72˚C for 45 s were repeated for 35 cycles and followed by completion at 72˚C for 4 min. The size of the expected PCR product was 414 bp. The PCR product was separated on 3% agarose gel, stained with ethidium bromide, and visualized under ultraviolet radiation. EZ Load™ (BIO-RAD, Tokyo, Japan) was used as a molecular ruler.

### RESULTS

### DC2.4 cells pulsed with OVA-Lipofectin efficiently present OVA-peptides on MHC class I molecules

In the present study, we used DC2.4 cells, a murine dendritic cell line (Shen et al, 1997), as APCs. Immunofluorescence analysis and flow cytometry confirmed the expression of MHC class I and II molecules and costimulatory molecules such as B7-1 (CD80), B7-2 (CD86), and CD40 on the surface of DC2.4 cells (data not shown). To investigate antigen presentation of OVA-pulsed DC2.4 cells via MHC class I molecules, we performed an antigen presentation assay by measuring the production of IL-2 from RF33.70 cells specific for OVA peptides bound to MHC class I molecules (Rock et al, 1990; Shen et al, 1997) (Figure 1). No significant change in IL-2 levels was observed in the co-cultures of soluble OVA-pulsed DC2.4 cells and RF33.70 cells, even when 10 mg/ml of soluble OVA was pulsed onto DC2.4 cells. On the other hand, when pulsed with OVA-Lipofectin (10 µg/ml), DC2.4 cells efficiently presented OVA-peptides on MHC class I molecules in an OVA concentration-dependent manner.

Because DC2.4 cells are an established cell line, the permanent proliferation of DC2.4 cells must be inhibited for in vivo vaccination. Therefore, we examined the effect of 50 µg/ml of MMC on the MHC class I presentation of DC2.4 cells pulsed with OVA-Lipofectin complexes. MMC-treatment did not affect the presentation of OVA-peptides via MHC class I molecules on DC2.4 cells (Figure 1). In addition, we confirmed that the MMC-treated DC2.4 cells maintained their viability without cell growth for at least 5 days in vitro, and did not show tumour formation when injected i.d. at $5 \times 10^6$ cells per mouse (data not shown). Taken collectively, these results suggest that MMC-treated DC2.4 cells pulsed with OVA-Lipofectin complexes may be useful for vaccination in subsequent experiments.

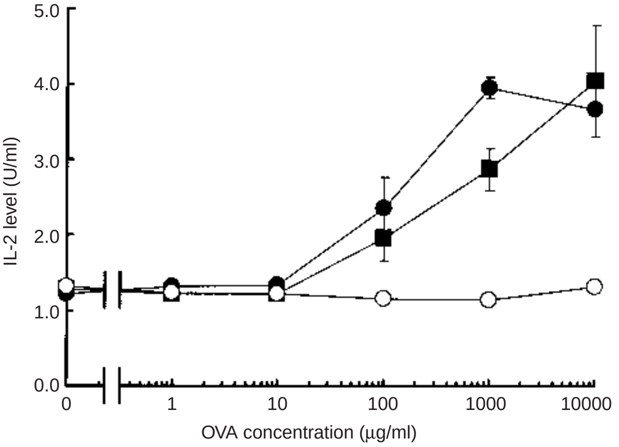

**Figure 1** Antigen presentation on MHC class I molecules by DC2.4 cells pulsed with soluble OVA or OVA-Lipofectin complexes. DC2.4 cells were incubated for 5 h at 37˚C with indicated concentrations of OVA in soluble form (○) or in complex with 10 µg/ml Lipofectin (●, ■). After washing, the cells were incubated in the presence (■) or the absence (○, ●) of 50 µg/ml of MMC at 37˚C for 30 min. Antigen presentation was determined by the CTLL-2 bioassay described in Materials and Methods. Each point represents the mean±SD of three independent cultures

### Protective effect against challenge with E.G7-OVA tumour cells varies with vaccination route

We next determined the effects of administration routes of antigen-loaded DCs on the efficacy of DC-based immunotherapy. As shown in Figure 2, C57BL/6 mice were immunized once with $1 \times 10^6$ OVA-pulsed DC2.4 cells by i.v., i.p., i.d., or s.c. injection. After inoculation with E.G7-OVA tumour cells on day 7 post-vaccination, the anti-tumour efficacy was found to vary based on the injection site of OVA-pulsed DC2.4 cells, although delay in tumour growth was observed in all immunized groups. No tumour formation was observed in the i.d.-and s.c.-injected groups or in three of four mice in the i.p.-injected group on day 21 post-tumour challenge. However, tumour formation was observed in all mice immunized by i.v. administration. Moreover, three of four mice in the i.d.-and s.c.-injected groups achieved complete rejection by day 60 after E.G7-OVA tumour challenge (Table 1). This anti-tumour

**Table 1** Summary of anti-E.G7-OVA tumour effects after immunization with OVA-pulsed DC2.4 cells

| Immunization: Schedule | Immunization: Route | Tumour volume[a] (mm³) | | | | Complete rejection[b] |
|---|---|---|---|---|---|---|
| Once | i.v. | 69.8 | 615.8 | 1028.7 | 2092.4 | 0/4 |
| | i.p. | 0.0 | 0.0 | 0.0 | 877.4 | 2/4 |
| | i.d. | 0.0 | 0.0 | 0.0 | 0.0 | 3/4 |
| | s.c. | 0.0 | 0.0 | 0.0 | 0.0 | 3/4 |
| Twice | i.v. | 0.0 | 0.0 | 978.7 | 1276.3 | 0/4 |
| | i.p. | 0.0 | 0.0 | 0.0 | 0.0 | 4/4 |
| | i.d. | 0.0 | 0.0 | 0.0 | 0.0 | 4/4 |
| | s.c. | 0.0 | 0.0 | 0.0 | 0.0 | 4/4 |
| | | 1341.6 | 1873.2 | >2500 | >2500 | 0/4 |

[a]Day 21 after tumour challenge. Tumour volume (mm³) = (major axis) × (minor axis)² × 0.5236. [b]Day 60 after tumour challenge, tumour-rejected mice/tumour-inoculated mice.

 *© 2001 Cancer Research Campaign*

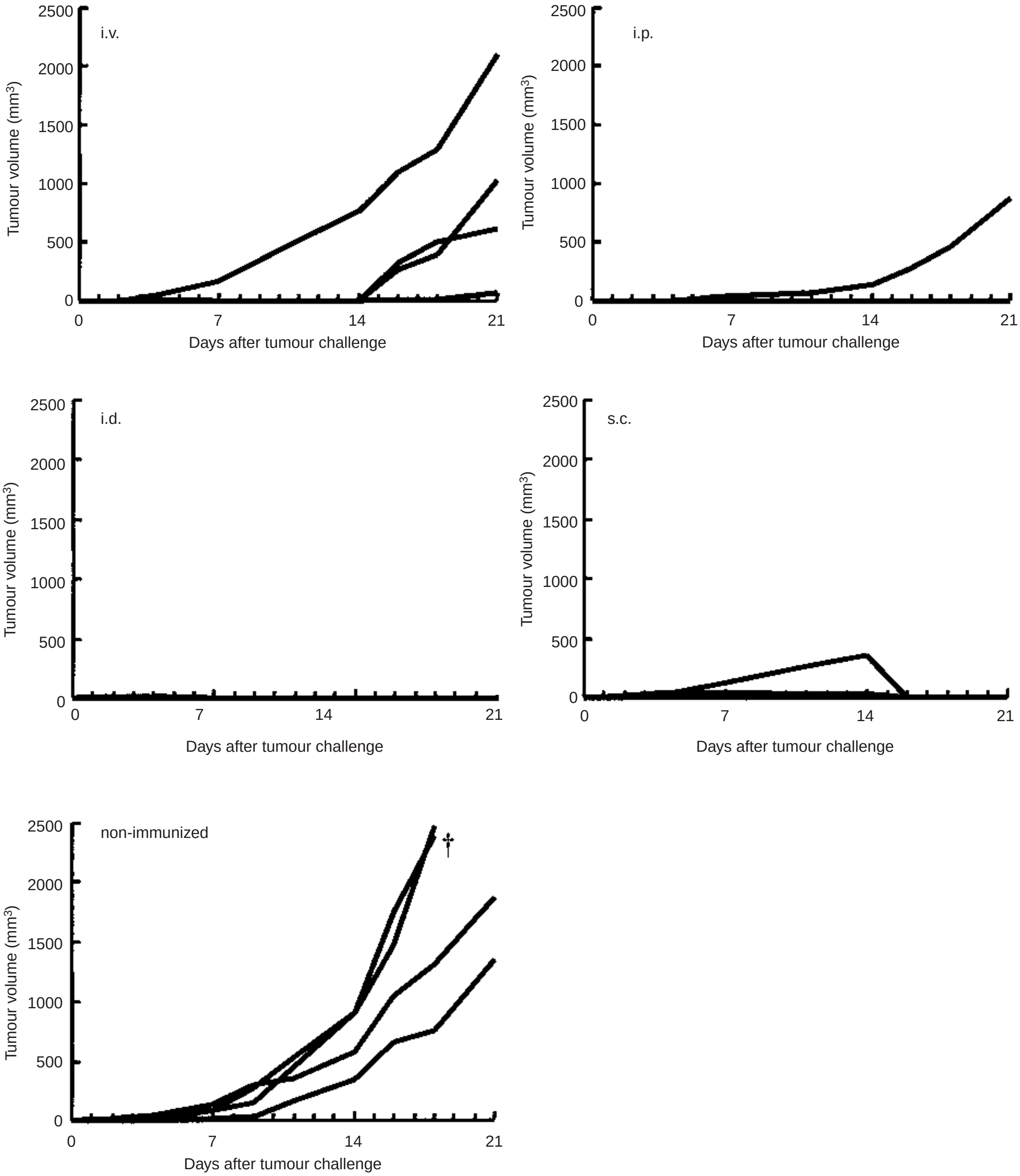

**Figure 2** Influence of administration route of OVA-pulsed DC2.4 cells on vaccine efficiency against E.G7-OVA tumour growth. C57BL/6 mice were immunized once via various routes by $1 \times 10^6$ DC2.4 cells pulsed with OVA (1 mg/ml)-Lipofectin. One week later, $1 \times 10^6$ E.G7-OVA tumour cells (OVA-transfectant of EL4 mouse thymoma cells) were intradermally inoculated into the left flank. The size of growing E.G7-OVA tumours was measured every 2–3 days using microcalipers. Each group consisted of four mice. †: Two mice were euthanized because tumour volumes were in excess of 2500 mm$^3$.

effect of OVA-pulsed DC2.4 cells was OVA-specific because after inoculation with EL4 tumour cells, parent cells of E.G7-OVA tumour growth was not affected by vaccination with OVA-pulsed DC2.4 cells (data not shown). Two vaccinations over a 1-week interval further potentiated the anti-tumour effect of all OVA-pulsed DC2.4 cell administration routes (Table 1). Complete protection against E.G7-OVA tumour challenge was achieved in all mice immunized twice with $1 \times 10^6$ OVA-pulsed DC2.4 cells via i.p., i.d., or s.c. route. Repeating the i.v. immunization failed to achieve complete rejection, although two of four mice were tumour-free on day 21 post-tumour challenge. These results demonstrated that the vaccine efficiency of DCs pulsed with antigens was obviously influenced by the administration route and the immunization schedule.

*© 2001 Cancer Research Campaign* 

### Administration routes of OVA-pulsed DC2.4 cells affect OVA-specific CTL response

Antigen-pulsed DC vaccines have been reported to trigger a specific CTL response directly (Paglia et al, 1996; Alters et al, 1998; Wong et al, 1998). We therefore used the Eu-release assay to determine the influence of OVA-pulsed DC2.4 cell administration route on the induction of the OVA-specific CTL response. Spleen cells were prepared from mice on day 7 after vaccination with OVA-pulsed DC2.4 cells via various routes. Cytotoxic effectors were generated by co-culturing spleen cells for 5 days with E.G7-OVA cells treated with MMC. As shown in Figure 3, strong lytic activity against E.G7-OVA cells was elicited in cultured spleen cells from mice administered OVA-pulsed DC2.4 cells i.d. or s.c. In contrast, moderate and low killing of E.G7-OVA cells was observed in the i.p.-and i.v.-immunized groups, respectively. Cytotoxic activity against EL4 cells and YAC-1 cells, which are highly sensitive to natural killer cells (Cikes et al, 1973), failed to be generated by vaccination with OVA-pulsed DC2.4 cells. This observation strongly suggested that the lysis of E.G7-OVA cells was due to the OVA-specific CTL activity. Collectively, these results demonstrate that the induction of antigen-specific CTL response is greatly affected by the administration route of the antigen-loaded DCs.

### Assessment of OVA-pulsed DC2.4 cell migration to lymphoid tissues

After activation by antigen challenge, DCs mature and migrate to the secondary lymphoid tissues in which they present the processed antigen to naïve T cells (Austyn et al, 1988; Rock, 1996). Thus, we investigated the migration of OVA-pulsed DC2.4 cells to the lymphoid organs in response to various administration routes. Since the DC2.4 cell line was reported to be established by infection with a retrovirus encoding *v-myc* and *v-raf* (Shen et al, 1997), we determined that the presence of DC2.4 cells in lymphoid tissues after vaccination could be detected by PCR using primer pairs specific for *v-myc*. Genomic DNA isolated from various lymphoid organs was subjected to PCR and the products were analyzed. In the case of i.d. administration of DC2.4 cells, DNA isolated from lumbal, pygal, inguinal, and ischial lymph nodes produced a PCR product of expected size (414 bp) (Figure 4, third panel). In the case of s.c. administration, the specific PCR product was amplified from lumbal, inguinal, and ischial lymph nodes (Figure 4, fourth panel). In contrast, genomic DNA from various lymphoid tissues failed to generate this PCR product after i.v. and i.p. administration of DC2.4 cells (Figure 4, first and second panels). These data show that i.d.-and s.c.-administered DC2.4 cells preferentially migrate to the regional lymph nodes, where DC2.4 cells interact with naïve T cells to prime an antigen-specific immune response. Therefore, the differential migration of OVA-pulsed DC2.4 cells following their administration by the i.v., i.p., i.d., or s.c. routes is reflected in their potency to induce OVA-specific CTL responses.

### DISCUSSION

DCs are professional APCs capable of capturing antigens and processing and presenting antigenic peptide fragments. Mature DCs can migrate to lymphoid organs to prime T cell immune responses (Austyn et al, 1988; Rock, 1996; Banchereau and Steinman, 1998). Due to these properties, DCs are considered promising tools for cancer immunotherapy. For successful DC-based immunotherapies, antigens must be efficiently introduced into DCs and presented on MHC class I molecules at high levels, to activate $CD8^+$ CTLs. We have been exploring methods for loading exogenous antigens into APCs showing high MHC class I presentation efficiency (Hayashi et al, 1999; Nakanishi et al, 2000). In the present study, we tested the cationic liposome, Lipofectin, for loading an exogenous antigen, OVA, into the murine DC2.4 cell line, which has been reported to possess morphological and functional properties of DCs (Shen et al, 1997). DC2.4 cells pulsed with OVA-Lipofectin complexes showed more efficient presentation of OVA-peptides on MHC class I molecules than soluble OVA-pulsed DC2.4 cells (Figure 1). In most cells, antigens in the extracellular fluids do not ordinarily access the MHC class I pathway for antigen presentation. In contrast, DCs have been reported to transfer the exogenous antigens from the endocytic compartment into the cytosol, where they are degraded and presented via the classical MHC class I pathway. This process is markedly enhanced when the antigens are internalized by phago-

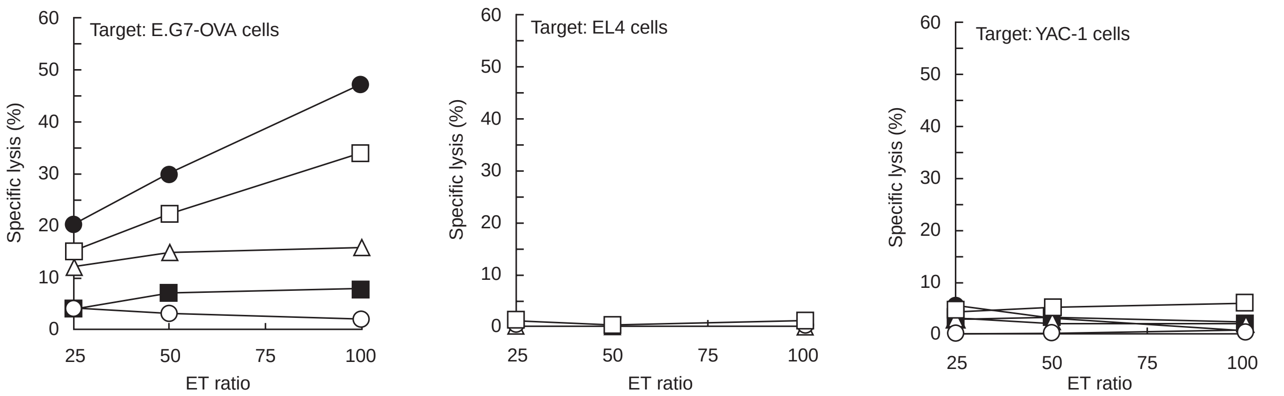

**Figure 3** OVA-specific CTL responses in mice immunized with OVA-pulsed DC2.4 cells via various routes. C57BL/6 mice were immunized by i.v. (■), i.p. (Δ), i.d. (●), and s.c. (□) injection with 1 $\times 10^6$ OVA-pulsed DC2.4 cells. Similarly, non-pulsed DC2.4 cells (O) were injected into mice intradermally. One week later, mice were sacrificed, their spleens removed and splenocytes were prepared. After in vitro restimulation with E.G7-OVA cells for 5 days, effector cells were incubated with Eu-labeled target cells (E.G7-OVA cells, EL4 cells, or YAC-1 cells) at 37˚C for 4 h. The specific lysis of target cells was determined by using the following formula; Specific lysis (%)=[(experimental Eu-release)-(spontaneous Eu-release)]/[(maximum Eu-release)-(spontaneous Eu-release)] $\times$ 100. Spontaneous release in the absence of effector cells was <10% of maximum release by detergent. All data points represent the average of two independent experiments, which were performed with 3–5 mice per group

*© 2001 Cancer Research Campaign*

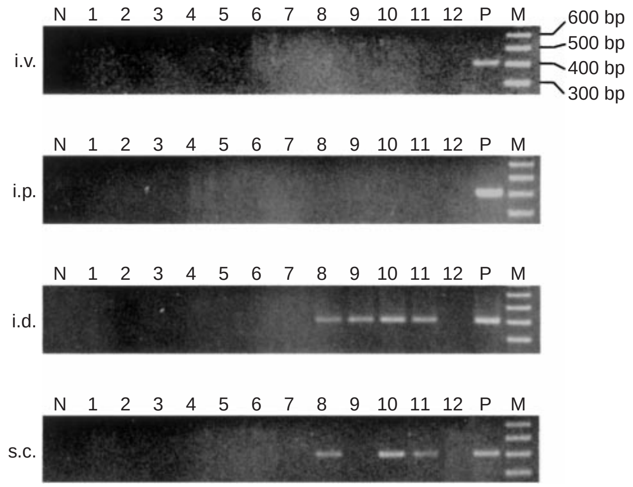

**Figure 4** PCR analysis of the migration to the lymphoid tissues of DC2.4 cells pulsed with OVA. C57BL/6 mice were injected via various routes with $1 \times 10^7$ OVA-pulsed DC2.4 cells. After 48 h, lymphoid tissues were removed and genomic DNA was isolated. PCR was performed using a primer pair specific for *v-myc*. Lane N is negative control run in parallel in the absence of DNA. Lanes 1–12 represent cervical lymph nodes, brachial lymph nodes, axillary lymph nodes, thymus, mesenterial lymph nodes, Peyer's patches, renal lymph nodes, lumbal lymph nodes, pygal lymph nodes, inguinal lymph nodes, ischial lymph nodes, and spleen, respectively. Lane P is a positive control using DC2.4 cells-derived genomic DNA. Lane M is a 100 bp-molecular ruler cytosis (Shen et al, 1997), macropinocytosis (Norbury et al, 1997), or receptor-mediated endocytosis (Rodriguez et al, 1999; Machy et al, 2000). In some cases, peptides derived from the exogenous antigens appear to be generated in the endocytic compartments and then bind to MHC class I molecules on the cell surface (Zitvogel et al, 1998). Although the pathway of internalized OVA-Lipofectin complexes in DC2.4 cells remains to be elucidated, we speculate that the transfer of OVA to the MHC class I pathway may be greatly facilitated by Lipofectin because DC2.4 cells used phagocytosis not pinocytosis to capture exogenous OVA associated with Lipofectin. Elucidation of intracellular trafficking of antigens loaded with Lipofectin and of the transport mechanism of the antigen-Lipofectin complex in DCs is important for establishing methods for efficient introduction of antigens into DCs showing high MHC class I presentation efficiency.

Several studies have shown that ex vivo-differentiated DCs may enable specific vaccination for generating protective tumour immunity in human clinical trials (Hsu et al, 1996; Nestle et al, 1998; Lodge et al, 2000). However, it is still unclear which DC administration route elicits superior therapeutic effects in DC-based immunotherapy. In order to elucidate this issue, we performed E.G7-OVA tumour rejection assays in mice immunized via various administration routes with DC2.4 cells pulsed with OVA-Lipofectin complexes. Although tumour growth was delayed in all groups, the ratio of complete rejection on day 60 after tumour challenge differed with the administration route of OVA-pulsed DC2.4 cells (Table 1). Moreover, the anti-tumour effect was enhanced in all groups by a twice repeated vaccination with OVA-pulsed DC2.4 cells. These results suggest that the route of administration and the immunization schedule of OVA-pulsed DC2.4 cells affect the induction of OVA-specific host immunity. In addition, these results are confirmed by the OVA-specific CTL response after vaccination with OVA-pulsed DC2.4 cells via various routes. High cytotoxicity due to OVA-specific CTLs was shown in splenocytes isolated from mice injected i.d. and s.c. with OVA-pulsed DC2.4 cells, whereas moderate and low CTL response was observed in i.p. and i.v. administered mice, respectively (Figure 3). The magnitude of OVA-specific CTL response induced by immunizing with OVA-pulsed DC2.4 cells, positively correlated with the results of the E.G7-OVA tumour rejection assay for each vaccination route.

The migration of antigen-loaded DCs to secondary lymphoid tissues is essential for efficient induction of the antigen-specific CTL response. Lappin et al (1999) reported that DCs were found in popliteal lymph nodes at 24 h following their s.c. injection into footpads. The number of DCs peaked at 48 h and decreased to background levels by day 5 after injection (Lappin et al, 1999). Based on their report, we investigated by PCR the migration of OVA-pulsed DC2.4 cells to the lymphoid tissues, 48 h after injection. The PCR product for *v-myc* was successfully amplified from genomic DNA isolated from the regional lymph nodes of i.d.-or s.c.-injected mice, while no PCR products were found in any lymphoid tissues of i.v.-or i.p.-injected mice (Figure 4). These results clearly indicate that the DC2.4 cells administered i.d. or s.c. preferentially migrated to the regional lymph nodes as compared with i.v.-and i.p.-injected DC2.4 cells. Therefore, we reasoned that the various anti-E.G7-OVA tumour effects of OVA-pulsed DC2.4 cells reflected the extent of their migration to the regional lymph nodes and thereby the induction of OVA-specific CTL response.

Recent advances in biological and immunological research on DCs have introduced the potential use of specific antigen-loaded DCs as nature's adjuvant against cancer. DC-based immunotherapy is expected to show strong anti-tumour effects against microscopic metastases that are otherwise incurable. However, further studies are required to improve the effectiveness of DC-based immunotherapy. In the present report, we provided evidence that the administration route affected the migration of antigen-loaded DCs to the regional lymph nodes and thereby dominated host tumour immunity based on the antigen-specific CTL response. These results demonstrate that the route of administration of DCs should be considered as an important variable when designing vaccination protocols using DCs loaded with antigens.

### ACKNOWLEDGEMENTS

We thank Dr Kenneth L. Rock (Department of Pathology, University of Massachusetts Medical School, MA, USA) for providing DC2.4 cells and RF33.70 cells, Dr Tsuyoshi Nakanishi (Department of Toxicology, Graduate School of Pharmaceutical Sciences, Osaka University) for performing the immunofluorescence and flow cytometry for DC2.4 cells, and Yoshihiro Fujii, Junko Fujita, Kyoko Fujimoto, and Yasushige Masunaga (Department of Biopharmaceutics, Kyoto Pharmaceutical University) for technical assistance in the PCR analysis.

Our work was supported in part by a Grant-in-Aid for Encouragement of Young Scientists (11771473) from the Japan Society for the Promotion of Science, and the Science Research Promotion Fund from the Japan Private School Promotion Foundation.

### REFERENCES

Alters SE, Gadea JR, Sorich M, O'Donoghue G, Talib S and Philip R (1998) Dendritic cells pulsed with CEA peptide induce CEA-specific CTL with restricted TCR repertoire. *J Immunother* **21**: 17–26

© 2001 Cancer Research Campaign 

Arthur JF, Butterfield LH, Roth MD, Bui LA, Kiertscher SM, Lau R, Dubinett S, Glaspy J, McBride WH and Economou JS (1997) A comparison of gene transfer methods in human dendritic cells. *Cancer Gene Ther* **4**: 17–25

Ashley DM, Faiola B, Nair S, Hale LP, Bigner DD and Gilboa E (1997) Bone marrow-generated dendritic cells pulsed with tumour extracts or tumour RNA induce antitumour immunity against central nervous system tumours. *J Exp Med* **186**: 1177–1182

Austyn JM, Kupiec-Weglinski JW, Hankins DF and Morris PJ (1988) Migration patterns of dendritic cells in the mouse. Homing to T cell-dependent areas of spleen, and binding within marginal zone. *J Exp Med* **167**: 646–651

Banchereau J and Steinman RM (1998) Dendritic cells and the control of immunity. *Nature* **392**: 245–252

Bernhard H, Disis ML, Heimfeld S, Hand S, Gralow JR and Cheever MA (1995) Generation of immunostimulatory dendritic cells from human $CD34^+$ hematopoietic progenitor cells of the bone marrow and peripheral blood. *Cancer Res* **55**: 1099–1104

Brossart P, Grunebach F, Stuhler G, Reichardt VL, Mohle R, Kanz L and Brugger W (1998) Generation of functional human dendritic cells from adherent peripheral blood monocytes by CD40 ligation in the absence of granulocyte-macrophage colony-stimulating factor. *Blood* **92**: 4238–4247

Celluzzi CM, Mayordomo JI, Storkus WJ, Lotze MT and Falo Jr LD (1996) Peptide-pulsed dendritic cells induce antigen-specific CTL-mediated protective tumour immunity. *J Exp Med* **183**: 283–287

Cikes M, Friberg Jr S and Klein G (1973) Progressive loss of H-2 antigens with concomitant increase of cell-surface antigen(s) determined by Moloney leukemia virus in cultured murine lymphomas. *J Natl Cancer Inst* **50**: 347–362

Eggert AA, Schreurs MW, Boerman OC, Oyen WJ, de Boer AJ, Punt CJ, Figdor CG and Adema GJ (1999) Biodistribution and vaccine efficiency of murine dendritic cells are dependent on the route of administration. *Cancer Res* **59**: 3340–3345

Fallarino F, Uyttenhove C, Boon T and Gajewski TF (1999) Improved efficacy of dendritic cell vaccines and successful immunization with tumour antigen peptide-pulsed peripheral blood mononuclear cells by coadministration of recombinant murine interleukin-12. *Int J Cancer* **80**: 324–333

Fearnley DB, McLellan AD, Mannering SI, Hock BD and Hart DN (1997) Isolation of human blood dendritic cells using the CMRF-44 monoclonal antibody: implications for studies on antigen-presenting cell function and immunotherapy. *Blood* **89**: 3708–3716

Gong J, Chen D, Kashiwaba M and Kufe D (1997) Induction of antitumour activity by immunization with fusions of dendritic and carcinoma cells. *Nat Med* **3**: 558–561

Harding CV (1994) Techniques for studying phagocytic processing of bacteria for class I or II MHC-restricted antigen recognition by T lymphocytes. *Methods Cell Biol* **45**: 313–326

Hayashi A, Nakanishi T, Kunisawa J, Kondoh M, Imazu S, Tsutsumi Y, Tanaka K, Fujiwara H, Hamaoka T and Mayumi T (1999) A novel vaccine delivery system using immunopotentiating fusogenic liposomes. *Biochem Biophys Res Commun* **261**: 824–828

Henkart PA (1994) Lymphocyte-mediated cytotoxicity: two pathways and multiple effector molecules. *Immunity* **1**: 343–346

Hsu FJ, Benike C, Fagnoni F, Liles TM, Czerwinski D, Taidi B, Engleman EG and Levy R (1996) Vaccination of patients with B-cell lymphoma using autologous antigen-pulsed dendritic cells. *Nat Med* **2**: 52–58

Janik P, Briand P and Hartmann NR (1975) The effect of estrone-progesterone treatment on cell proliferation kinetics of hormone-dependent GR mouse mammary tumours. *Cancer Res* **35**: 3698–3704

Kronenberg M, Siu G, Hood LE and Shastri N (1986) The molecular genetics of the T-cell antigen receptor and T-cell antigen recognition. *Annu Rev Immunol* **4**: 529–591

Lappin MB, Weiss JM, Delattre V, Mai B, Dittmar H, Maier C, Manke K, Grabbe S, Martin S and Simon JC (1999) Analysis of mouse dendritic cell migration in vivo upon subcutaneous and intravenous injection. *Immunology* **98**: 181–188

Lodge PA, Jones LA, Bader RA, Murphy GP and Salgaller ML (2000) Dendritic cell-based immunotherapy of prostate cancer: immune monitoring of a phase II clinical trial. *Cancer Res* **60**: 829–833

Ludewig B, Ochsenbein AF, Odermatt B, Paulin D, Hengartner H and Zinkernagel RM (2000) Immunotherapy with dendritic cells directed against tumour antigens shared with normal host cells results in severe autoimmune disease. *J Exp Med* **191**: 795–803

Lutz MB, Kukutsch N, Ogilvie AL, Rossner S, Koch F, Romani N and Schuler G (1999) An advanced culture method for generating large quantities of highly pure dendritic cells from mouse bone marrow. *J Immunol Methods* **223**: 77–92

Machy P, Serre K and Leserman L (2000) Class I-restricted presentation of exogenous antigen acquired by Fcgamma receptor-mediated endocytosis is regulated in dendritic cells. *Eur J Immunol* **30**: 848–857

Mayordomo JI, Zorina T, Storkus WJ, Zitvogel L, Celluzzi C, Falo LD, Melief CJ, Ildstad ST, Kast WM, Deleo AB and Lotze MT (1995) Bone marrow-derived dendritic cells pulsed with synthetic tumour peptides elicit protective and therapeutic antitumour immunity. *Nat Med* **1**: 1297–1302

Mosmann T (1983) Rapid colorimetric assay for cellular growth and survival: application to proliferation and cytotoxicity assays. *J Immunol Methods* **65**: 55–63

Muller G, Muller A, Jonuleit H, Steinbrink K, Szalma C, Paragnik L, Lingnau K, Schmidt E, Knop J and Enk AH (2000) Fetal calf serum-free generation of functionally active murine dendritic cells suitable for in vivo therapeutic approaches. *J Invest Dermatol* **114**: 143–149

Nair S, Zhou F, Reddy R, Huang L and Rouse BT (1992) Soluble proteins delivered to dendritic cells via pH-sensitive liposomes induce primary cytotoxic T lymphocyte responses in vitro. *J Exp Med* **175**: 609–612

Nair SK, Snyder D, Rouse BT and Gilboa E (1997) Regression of tumours in mice vaccinated with professional antigen-presenting cells pulsed with tumour extracts. *Int J Cancer* **70**: 706–715

Nakanishi T, Hayashi A, Kunisawa J, Tsutsumi Y, Tanaka K, Yashiro-Ohtani Y, Nakanishi M, Fujiwara H, Hamaoka T and Mayumi T (2000) Fusogenic liposomes efficiently deliver exogenous antigen through the cytoplasm into the MHC class I processing pathway. *Eur J Immunol* **30**: 1740–1747

Nestle FO, Alijagic S, Gilliet M, Sun Y, Grabbe S, Dummer R, Burg G and Schadendorf D (1998) Vaccination of melanoma patients with peptide-or tumour lysate-pulsed dendritic cells. *Nat Med* **4**: 328–332

Norbury CC, Chambers BJ, Prescott AR, Ljunggren HG, Watts C (1997) Constitutive macropinocytosis allows TAP-dependent major histocompatibility complex class I presentation of exogenous soluble antigen by bone marrow-derived dendritic cells. *Eur J Immunol* **27**: 280–288

Paglia P, Chiodoni C, Rodolfo M and Colombo MP (1996) Murine dendritic cells loaded in vitro with soluble protein prime cytotoxic T lymphocytes against tumour antigen in vivo. *J Exp Med* **183**: 317–322

Philip R, Alters SE, Brunette E, Ashton J, Gadea J, Yau J, Lebkowski J and Philip M (2000) Dendritic cells loaded with MART-1 peptide or infected with adenoviral construct are functionally equivalent in the induction of tumour-specific cytotoxic T lymphocyte responses in patients with melanoma. *J Immunother* **23**: 168–176

Rock KL, Rothstein L and Gamble S (1990) Generation of class I MHC-restricted T-T hybridomas. *J Immunol* **145**: 804–811

Rock KL (1996) A new foreign policy: MHC class I molecules monitor the outside world. *Immunol Today* **17**: 131–137

Rodriguez A, Regnault A, Kleijmeer M, Ricciardi-Castagnoli P and Amigorena S (1999) Selective transport of internalized antigens to the cytosol for MHC class I presentation in dendritic cells. *Nat Cell Biol* **1**: 362–368

Romani N, Gruner S, Brang D, Kampgen E, Lenz A, Trockenbacher B, Konwalinka G, Fritsch PO, Steinman RM and Schuler G (1994) Proliferating dendritic cell progenitors in human blood. *J Exp Med* **180**: 83–93

Roskrow MA, Dilloo D, Suzuki N, Zhong W, Rooney CM and Brenner MK (1999) Autoimmune disease induced by dendritic cell immunization against leukemia. *Leuk Res* **23**: 549–557

Shen Z, Reznikoff G, Dranoff G and Rock KL (1997) Cloned dendritic cells can present exogenous antigens on both MHC class I and class II molecules. *J Immunol* **158**: 2723–2730

Steinman RM (1991) The dendritic cell system and its role in immunogenicity. *Annu Rev Immunol* **9**: 271–296

Tillman BW, de Gruijl TD, Luykx-de Bakker SA, Scheper RJ, Pinedo HM, Curiel TJ, Gerritsen WR and Curiel DT (1999) Maturation of dendritic cells accompanies high-efficiency gene transfer by a CD40-targeted adenoviral vector. *J Immunol* **162**: 6378–6383

Volgmann T, Klein-Struckmeier A and Mohr H (1989) A fluorescence-based assay for quantitation of lymphokine-activated killer cell activity. *J Immunol Methods* **119**: 45–51

Wong C, Morse M and Nair SK (1998) Induction of primary, human antigen-specific cytotoxic T lymphocytes in vitro using dendritic cells pulsed with peptides. *J Immunother* **21**: 32–40

Zitvogel L, Regnault A, Lozier A, Wolfers J, Flament C, Tenza D, Ricciardi-Castagnoli P, Raposo G and Amigorena S (1998) Eradication of established murine tumours using a novel cell-free vaccine: dendritic cell-derived exosomes. *Nat Med* **4**: 594–600

© 2001 Cancer Research Campaign
